# Indian research on suicide

**DOI:** 10.4103/0019-5545.69255

**Published:** 2010-01

**Authors:** Lakshmi Vijayakumar

**Affiliations:** Sneha, Voluntary Health Services, 25/21 Ranjit Road, Kotturpuram, Chennai - 600 085, India

**Keywords:** Suicide, India, Risk factors

## Abstract

The suicide rate in India is 10.3. In the last three decades, the suicide rate has increased by 43% but the male female ratio has been stable at 1.4 : 1. Majority (71%) of suicide in India are by persons below the age of 44 years which imposes a huge social, emotional and economic burden. Fifty four articles on suicides have been published in IJP. Several studies reveal that suicidal behaviours are much more prevalent than what is officially reported. Poisoning, hanging and self immolation (particularly women) were the methods to commit suicide. Physical and mental illness, disturbed interpersonal relationships and economic difficulties were the major reasons for suicide. The vulnerable population was found to be women, students, farmers etc. A social and public health response in addition to a mental health response is crucial to prevent suicidal behaviour in India.

## INTRODUCTION

More than one lakh lives are lost every year due to suicide in India. In the last three decades (from 1975 to 2005), the suicide rate increased by 43%. The rates were approximately the same in 1975 and 1985; from 1985 to 1995 there was an increase of 35% and from 1995 to 2005, the increase was 5%. However, the male-female ratio has been stable at around 1.4 to 1. There is a wide variation in suicide rates within the country. The southern states of Kerala, Karnataka, Andhra Pradesh and Tamil Nadu have a suicide rate of >15 while in the Northern States of Punjab, Uttar Pradesh, Bihar and Jammu and Kashmir, the suicide rate is <3. This variable pattern has been stable for the last 20 years. Higher literacy, a better reporting system, lower external aggression, higher socioeconomic status and higher expectations are the possible explanations for the higher suicide rates in the southern states (Vijayakumar L, 2008).[1]

Majority of the suicides (37.8%) in India are by those below the age of 30 years. The fact that 71% of suicides in India are by persons below the age of 44 years imposes a huge social, emotional and economic burden on society.

The near equal suicide rates of young men and women and consistently narrow male:female ratio denotes that more Indian women die by suicide than their Western counterparts. Poisoning (34.8%), hanging (31.7%) and self-immolation (8.5%) were the common methods used to commit suicide (accidental deaths and suicide 2007).[2] Two large epidemiological verbal autopsy studies in rural Tamil Nadu reveal that the annual suicide rate is six to nine times the official rates. If these figures are extrapolated it suggests that there are at least half a million suicides in India every year. It is estimated that one in 60 persons are affected by suicide. It includes both, those who have attempted suicide and those who have been affected by the suicide of a close family or friend. Thus, suicide is a major public and mental health problem which demands urgent action.

Fifty four articles on “Suicide” have been published in the IJP from 1958 to 2009. The relative paucity in publications can be attributed to several factors but chiefly to the fact that it is an extremely difficult area to take up for research considering its sensitive nature, associated stigma and legal implications. It is interesting to note that the first article on attempted suicide appeared only in 1965. The articles ranged from references to suicide in ancient literature to psychobiological variables in suicide, epidemiological studies to prevention strategies.

The publications have been categorized under (1) Incidence and prevalence studies (2) Profiling and identification of risk factors (3) Suicide and suicidal behavior in specific communities (4)Studies on Non Fatal Deliberate Self Harm (DSH) (5) Suicide prevention strategies (6) and other, suicide related publications. The segregation is for the sake of convenience alone and should not be seen as being exclusive to its allocated category.

There have been four studies from abroad published in the IJP that have not been covered in the present review, these comprise of a study on women from Trinidad and Tobago, a study from US on adolescence, on teenage suicide attempters from UK and a Japanese study on pesticide suicides.

## INCIDENCE AND PREVALENCE STUDIES

There have been several studies reporting the incidence of suicide in India. Over the years the studies have reported incidence rates ranging from 2.36 to 42 per 100,000 populations. The majority of these have been hospital based studies along with a few community based samples.

In one of the first article on attempted suicide published by the IJP, Venkoba Rao[3] reported an incidence rate of 43 / 100,000 in Madurai. He also reported that 1 in 12 cases of suicide attempts were fatal.

Nandi *et al*.[4] studied incidence rates in Bengal using data available in the public domain across a hundred year period (1872-1972) and reported that the incidence of suicide had increased significantly from 2.36/100,00 in 1872 to 15.96 in 1972. The study also revealed that there was preponderance of male suicides, the vulnerable age group being those between the ages of 18 to 30 and the most common method employed was poisoning.

Hedge[5] in his study on the patterns of suicide in a rural community in northern Karnataka reported an incidence rate of 9.3/100,000. The study also reported a male (67%) preponderance. The study also revealed that rural suicide patterns did not vary from urban.

In contrast to these reports Shukla *et al*.[6] in their study on the incidence of suicides in Jhansi city reported more suicides among women (34 / 100,000) than men (24 / 100,000). Several other gender related differences were also reported, women were significantly younger (24.6 years) compared to the men (28.9 years), self immolation was the most frequent method of suicide by women while for men it was being run over by a train. Domestic strife and mental illness were identified as the most common causative factors. The study reported an incidence rate of 29/100,000.

These findings were supported by Banarjee *et al*.[7] who studied the vulnerability of Indian women. They found that the incidence of suicide was 43/100,000 in Bengal and that women (79.3%) outnumbered men. 75% of the victims were below 25 years of age and the commonest cause for suicide in women was quarrel with husband, while in men it was with parents. Ingestion of insecticide was the most common method of committing suicide.

## PROFILING AND IDENTIFICATION OF RISK FACTORS

Majority of the published studies on suicide have dealt with identifying the socio-demographic and psychosocial aspects of suicide attempters and those who have completed suicide. Some of these have also attempted to identify the characteristic differences between the two groups. Most of these were hospital based studies. The study methods used varied, from use of psychological autopsies to interviews to perusal of records.

Venkoba Rao[3] in his hospital based study on suicide attempts reported a preponderance of males and identified the vulnerable age group as being those from 15 to 25 years. Lack of social cohesion was identified as a significant risk factor. 20% of the attempters also had a family history of mental illness/suicidal attempts. The method of attempting suicide as well as the time (during daytime or night), were not seen as factors influencing intent.

In another hospital based study Lal and Sethi[8] reported that women attempted suicide more often, were below 30 years of age, were housewives or domestic help, married and income levels of 83.4% was less or equal to Rs. 200 per month. Females with lower educational level and joint families and males with higher educational levels and from unitary families attempted suicide more frequently. Similarly, a study by Badrinarayana[9] also revealed that younger people (age range of 10 to 30 years) were more likely to attempt suicide. The primary causes were identified as Mental illness and disturbed interpersonal relationships. Extramarital affair was also identified as a risk factor for a spouse to attempt suicide by Venkoba Rao.[10]

Nandi *et al*.[11] investigated the relationship between availability of lethal insecticide and the incidence of suicide. The study concluded that there was no association between the easy availability of the lethal insecticide and the high incidence of suicide but rather it was the motive which actually determines the incidence of suicides.

Bagadia *et al*.[12] attempted to examine the relationship between unemployment and suicide and concluded that though unemployment may be an important factor in suicide it did not appear to be the causative factor. The study postulated that both unemployment and suicidal behavior could be due to some common psychopathological factors. However, Srivatsava *et al*.[13] (2004) identified unemployment, presence of a stressful life event in the last six months, suffering from physical disorders and having idiopathic pain as definite risk factors for attempting suicide.

In their study from Ludhiana, Narang *et al*.[14] reported that single males and married females were more likely to attempt suicide. They, however, did not find type of family, economic status and educational levels as being significant variables. Mood disorders and adjustment disorders were diagnosed in a significant number of them.

Bagadia *et al*.[15] conducted a study on 521 patients admitted for suicidal behavior and reported that the degree of intent was low, duration of suicidal ideas ranged from more than 1 year (2%) to it being an impulsive act in 17% of them, 18% communicated about the attempt while the majority of women (76.1%) attempted suicide in the presence/proximity of others. Previous attempts were reported in 7% with 2.4% having more than one previous attempt. Depression (39.73%), schizophrenia (24.4%) and hysteria (14%) were the most common psychiatric diagnosis made.

These findings were also confirmed by Gupta and Singh[16] who reported psychiatric disorders in 62% with 58% having abnormal personalities. Mahla, *et al*.[17] investigated attempted cases of self immolation and reported that the behavior was associated with the presence of psychiatric and personality disorders. Jain, *et al*.[18] also found that 37.5% of the suicide attempters had a diagnosis of depression, 39.28% of the subjects showed mild to moderated suicidal intent and 16% of them had a high score on the hopelessness variable. Similarly, in their study using the method of psychological autopsy, Khan, *et al*.[19] identified the presence of psychiatric illness and stressful life events as the two most important reasons for completing suicide.

Badrinarayana[20] found a positive and significant correlation between depressive illness, suicidal ideation with early parental deprivation, recent bereavement and positive family history of suicide. Similarly Srivastava and Kulshreshtha[21] reported a positive correlation between severity of depression, being married, being employed, being male, prior history of treatment in a mental hospital setting, more than a month’s duration of illness and age being less than or equal to 35 years.

Anand, *et al*.[22] in their study on suicidal intent identified three distinct groups comprising of non communicators (31.9%), partial communicators (32.6%) and definite communicators (35.5%). A study by Ponnudurai *et al*.[23] revealed that 23.25% had contemplated suicide earlier and that 91.9% of them were aged 30 years or less. A strong association with alcohol was reported in 10.42% of the sample.

In his comparison study between suicide attempters and completers, Suresh Kumar,[24] reported that those who completed suicide were significantly younger, they were more frequently unemployed and used more lethal methods (hanging) than those who attempted. Other variables such as religion, domicile, marital status and education showed no difference.

Very few studies pertaining to the biology of suicides have been published in the IJP. The earliest article published was by Devi and Rao,[25] who studied the association between suicide attempts and menstrual cycles. The study reported that women in their pre-menstrual/early menstrual phase (64%) were more vulnerable. Marital status of the patients did not contribute to any heightened vulnerability during premenstruum and menstruation.

Palaniappan, *et al*.[26] explored the possible association between suicidal ideation and biogenic amines. They observed that the levels of 5 HIAA and Serotonin (5HT) were inversely related to suicidal ideas. Rao and Devi[27] in their article state that evidence from genetic research, mono amine studies and psychopharmacological research points towards a possible biological predisposition and precipitant for suicidal behavior.

## SUICIDE AND SUICIDAL BEHAVIOR IN SPECIFIC COMMUNITIES

There have been several studies which focus on vulnerable populations and high risk populations including students, the aged, women, armed forces, farmers, migrant populations and those with chronic physical and mental illness.

Venkoba Rao,[28] in his article on attempted suicide among students, reported that during a 10-month period 35 students had attempted suicide, of which seven proved fatal. The most common mode was insecticide ingestion. There were more male students (19) than female (16), most were aged between the ages of 16 to 30 and majority of them were students of Arts and Sciences. Eight of them had attempted suicide previously. No intellectual sub-normality was reported in the sample.

In an another study on the psychosocial and clinical factors associated with adolescent suicidal attempts Kumar, Sudhir *et al*.[29] compared potential risk factors between adolescent and adult suicide attempters and found that the adolescents had significantly higher levels of depression, hopelessness, lethality of event, and stressful life events. Sharma, *et al*.[30] in their study on adolescent students found the prevalence of suicide risk behavior quite high with almost 16% having suicide ideation and 5% having attempted suicide. Females were seen as being more vulnerable. The presence of role models who were seen drinking and smoking was seen as increasing the risk behavior.

Rao Venkoba,[31] studied depression and suicidal behavior in the aged and reported that the risk of completed suicide among the aged attempters is twice that of the younger generation. He also identified lack of social integration rather than social isolation per se as the factor causing depression in the aged.

In a study on 100 female burns cases admitted at the Madurai Medical college Venkoba Rao, *et al*.[32] reported that that 70% of them were suicidal attempts, 25% were accidental, 3% were homicidal and 2% were non classifiable. The most common reasons for suicidal attempts were marital and interpersonal problems followed by psychiatric and physical illnesses respectively.

Jacob, *et al*.[33] in their comparative study on subjects with seizure disorder and bronchial asthma found that 34% of the epilepsy group had a diagnosis of major depressive disorder as compared to 13.3% of the asthma group. Sixteen per cent of the epilepsy group had a history of at least one suicidal attempt in the previous year and 20% of the group expressed current suicidal ideation.

In a study on terminally ill cancer patients Latha and Bhat[34] examined the prevalence of suicidal ideation and reported that only 9.2 % had severe suicidal ideations. 3.8% of the patients with suicidal ideation had a past history of major depression. Factors such as presence of pain, awareness of diagnosis, and understanding of the illness contributed to the depressive states. The study concluded that suicidal ideation and desire for death appeared to be linked exclusively to the presence of a psychiatric disorder.

Satyavati[35] investigated attempted suicides in psychiatric in patients and reported that during a one year period out of 1881 admissions 126 had made suicidal attempts with drowning being the most commonly employed method. Patients with schizophrenia accounted for 64% of the attempted suicides. Gupta, *et al*.[36] in their two-year follow-up study of patients who had attempted suicide with schizophrenia and depression reported that 51.8% of the suicide attempters had a personality disorder, 42% had neurotic symptoms during childhood and 23.5% had a history of drug dependence. During the follow-up period 17.1% of the schizophrenia patients had attempted suicide again with one completing suicide, compared to 19% of the depressed patients.

Srivastava and Kumar[37] in their study on patients with major depressive disorder reported that the 17% in patients with suicidal ideation attempted suicide, The risk factors identified were being below 30 years of age, having higher education, being a single male or a married woman or a student. Suicide attempters also had more suicidal ideation, agitation and paranoid symptoms.

In a study on the armed forces Goel[38] argued that suicidal attempts do not constitute a major health concern in the army and that being in the army does not make the individual more vulnerable than the general population to suicide. Chakraborthy[39] in his study reported that the age of suicide attempters in the Army in India was higher than those reported from western countries. Isolation and inability to form relationships were identified as important factors in the suicidal attempts.

The need to focus on migrants as a specifically vulnerable group was brought out by the study of Chavan, *et al*.[40] who used psychological autopsies to reveal that almost 58% were migrants from other parts of India, were frequently male and young (age 20 to 28 years). Hanging was the most commonly used method for committing suicide. Psychosocial stressors were found in 61% and psychiatric illness was found in 34%. Only 16% had sought treatment prior to their attempt

On a study on farmer suicides in the Vidarbha region, Behere and Behere[41] employed the psychological autopsy method to understand the phenomenon and have identified the following reasons for farmer suicide (1) chronic indebtedness and inability to pay debts accumulated over the years (2) economic decline that leads to complications, family disputes, depression, alcoholism, etc. (3) compensation following suicide helps the family repay debts (4) grain drain and (5) rising costs of agricultural inputs and falling prices of agricultural produce.

## STUDIES ON NON FATAL DELIBERATE SELF HARM

Sethi, *et al*.[42] studied 75 patients admitted for self destructive behavior and found that majority of them belonged to unitary family set up, were unmarried males and almost 15% of them had history of previous suicidal attempts. Financial stress, rejection in love and strained familial relationships were the most common causes.

Sarkar *et al*.[43] attempted to present a profile of those who commit DSH in comparison with those who expected to die after the suicide attempt. Those attempting DSH were younger, chose less lethal methods to attempt suicide, were more impulsive and had strong histrionic and unstable traits in personality and had an absence of a family history of suicide attempts.

Das, *et al*.[44] in their study on subjects with intentional self harm attempts reported that the majority of the subjects were married, educated beyond matriculation, were employed or retired, belonged to a nuclear family, were of a middle socio economic status, and came from an urban background. The most common reasons for the attempt were interpersonal problems with family members and spouse. The most common mode was consumption of insecticides followed by use of corrosives. The most common psychiatric diagnosis in the group was depression. The use of organophosphorous pesticide poisoning for DSH was also reported by Chowdhury *et al*.[45] who found it the most commonly used method.

In their study on non fatal deliberate self harm attempters, Chowdhury *et al*.[46] identified women exposed to domestic violence as a vulnerable group. They were generally below 30 years of age, married and with low education, Pesticide poisoning was the commonest mode of DSH attempt. Marital conflicts, conflicts with in-laws were the typical stressors. Majority of them experienced more than one form of domestic violence. The study concluded that stressful life situations along with easy availability of pesticides facilitated self harm behavior.

## SUICIDE PREVENTION STRATEGIES

There have been very few articles that have dealt exclusively with suicide preventive strategies or with a scientific and systematic evaluation of a strategy.

Singh[47] in his article evaluated the various suicide prevention activities such as the community activities, the psychiatric and medical activities, suicide prevention centers, psychiatric emergency services, crises intervention centers, role of general practitioners, research and media. He concluded by stressing on the role of the psychiatrist in dealing with this issue.

Venkoba Rao[48] in his article delineated the risk factors associated with suicidal attempts and its association with psychiatric disorders and the biological evidence for suicidal behavior. The article based on cited studies recommended that education of general physicians, limiting access to availability of antidepressants, paracetemol and pesticides would lower the rates of suicide.

Jena and Siddharta[49] reviewed articles on non fatal suicidal attempts of adolescents in both Indian and international literature. They stated that non fatal suicidal behavior among adolescents needs to be evaluated and managed effectively in order to reduce the rates. They concluded that Indian studies in this area are a very few and there is a great need to conduct research in this area. The article also stresses the importance for professionals like general practitioners, teachers, pediatricians, school counselors to be trained to identify non fatal suicide behaviors in adolescents so as to facilitate referral and effective management.

Vijayakumar[50] in an editorial expresses the urgent need for suicide prevention in India and stresses that suicide is a multifaceted problem and hence suicide prevention programs should also be multidimensional. Collaboration, coordination, cooperation and commitment are needed to develop and implement a national plan, which is cost-effective, appropriate and relevant to the needs of the community. In India, suicide prevention is more of a social and public health objective than a traditional exercise in the mental health sector. She concludes by saying that the time is ripe for mental health professionals to adopt proactive and leadership roles in suicide prevention and save the lives of thousands of young Indians.

## OTHER SUICIDE RELATED PUBLICATIONS

Gupta, *et al*.[51] published an article on the development of a 10 item suicidal intent questionnaire. The article established that the questionnaire was fairly valid but stated that further work was necessary to establish its statistical validity and reliability.

Somasundaram *et al*.[52] in their paper described the presence of suicide behavior as found in ‘Purananuru’ an ancient Tamil classic from the ‘Sangham’ period. The article documents the self immolation of Perun Koppendu on the death of her husband, the fast unto death of a Cheran king in response to being insulted by guards and suicides of important kings and poets because of bereavement. The influence of religion and other cultural beliefs and its influence on perceptions of suicide and its representation in popular culture with specific referenced Tamil literary classics has been brought out in this article.

## CONCLUSION

A social and public health response to suicide is crucial in India, and should complement a mental health response. Mental illness is a risk factor for suicide, in India, as it is in developed countries. However, additional risk factors are prominent in India. These tend to relate to societal structures and specific stressors. A social and public health approach acknowledges that suicide is preventable, and promotes a framework in integrated system of interventions across multiple levels within society including the individual, the family, the community, and the health care system. A key step in such an approach involves modifying attitudes toward suicide via educational efforts and legal levers (e.g. decriminalizing suicide).
